# Inside-out anal sacculectomy in small dog breeds and cats

**DOI:** 10.3389/fvets.2023.1105826

**Published:** 2023-03-09

**Authors:** Sang Gwan Lee, Seong Mok Jeong, Sunhee Bae, Yeonhee Park, Changhwan Moon, Hee Young Kim

**Affiliations:** ^1^Joeun Animal Medical Center, Daegu, Republic of Korea; ^2^Department of Veterinary Surgery, College of Veterinary Medicine, Chungnam National University, Daejeon, Republic of Korea; ^3^Department of Physiology, College of Medicine, Yonsei University, Seoul, Republic of Korea

**Keywords:** anal sacculectomy, dog, cat, inside-out, surgery

## Abstract

This report describes a new, simple and rapid surgical technique for the removal of anal sac in small dogs and cats. The anal sacs were simply everted using mosquito hemostatic forceps and excised with the aid of an electrocautery surgical unit. On the evaluation of postoperative complications, only one dog of 28 animals experienced short-term minor complications of mild fecal incontinence and scooting. Thus, we suggest that this new surgical technique is easy, inexpensive and time-saving and some of the complications with previously reported methods used for small dog breeds and cats may be avoided by using this technique.

## Introduction

The anal sacs in dogs and cats are located underneath the external anal sphincter fibers and consist of two sinuses adjacent to the anus in dogs and cats (1). The anal sac ducts open on the lateral margin of anus at the anocutaneous junction, at the 4- and 8-o'clock positions. The sacs are connected to the anus by small ducts, produce a fluid, perceived as malodorous. While the glands normally release secretions during defecating, some animals suffer from anal sac impaction, chronic anal sacculitis, or anal sac abscesses (2). The most common cause for anal sac disorders is anal sacculitis, although the pathophysiology is not entirely understood (3). Medical management of anal sacculitis involves manual expression of the anal sacs, increasing dietary fiber or topical or systemic therapy with antibiotics and/or corticosteroids (4). Patients often do not respond satisfactorily to medical management or signs recur after initial response. Such inflammatory lesions can cause major problems and may reduce the quality of life of dogs and cats, especially as in-house pets. Permanent removal of the anal sacs is often recommended to treat recurrent or persistent anal sac disease (2, 5).

Anal sacculectomy is the surgical process of removing the anal glands (2, 5). Three conventional surgical techniques of the anal sacculectomy have been described, depending on whether the sac is or is not opened during dissection; these include standard open, modified open, or closed surgical techniques (5–7). Standard or modified open techniques incise the skin and anal sac for removal of anal sac. This technique provides visualization of the lining of the anal sacs and enables complete removal of the anal sac and its duct. However, there are disadvantages including iatrogenic trauma to the external anal sphincter muscles and higher risk of perioperative infection from contamination by contents of anal sacs (1, 6). With the closed technique, the anal sac is excised intact, aseptically and without incising its lumen. Although the closed technique is less likely to damage the external anal sphincter muscles and contaminate surrounding tissues, compared to the open technique, in some cases it can be difficult to identify the anal sac wall and complete removal of anal sac, especially in cases of severe anal sacculitis (7). A modified closed approaches have been introduced (1, 8). For example, a 6-French Foley catheter is placed into the anal sacs through duct and the balloon is inflated with saline solution to better visualize the anal sac after an incision made on the skin over the dilated anal sac during balloon inflation (8).

Open and closed surgical techniques for removal of anal sacs have been known to be safe and effective methods for non-neoplastic cases (6). Major surgical complication rate was reported as low and the incidence of minor postoperative complication rates is 3.2% to 32.3% (6). These include short-term (i.e., excessive drainage, scooting and inflammation) and long-term complications (i.e., fecal incontinence and stricture formation) (6). In a retrospective study of 95 dogs, the open technique causes more short- and long-term complications than the closed technique (6). Another retrospective study, in which the closed technique was applied bilaterally, reported that smaller (<15 kg) dogs are more likely to experience postoperative complications (9). Complications of the anal sacculectomy can be lowered when surgery is carried out after appropriate medical management (10).

The present report describes a new surgical technique for anal sacculectomy that can be simple, rapid, and may reduce postoperative complications in dogs and cats ranging in size from 2 to 10 kg.

## Materials and methods

### Animals

Twenty-eight client-owned animals with recurrent anal sac impaction (16 dogs and 11 cats) or inflammation (1 dog) were admitted to the Joeun Animal Medical Center (Daegu, Korea) for anal sacculectomy. Owners of all animals signed an informed consent form for this surgery. The animals underwent a comprehensive physical examination, followed by laboratory examination consisting of complete blood count (CBC) and a serum biochemistry panel.

### Study population and inclusion criteria

The medical records of dogs and cats that received anal sacculectomy were reviewed and included in this study. The dogs were excluded from the study if the dog did not show clinical problems in anal sacs such as recurrent anal sac impaction and infection. Information obtained through the medical record included patient identification number, blood examinations, admission, owner, clinician, weight, type of anal sac disease, age at the time of surgery, discharge date, sex, castration status, species, breed, medications, surgical time and complications. Dogs and cats without medical records about postoperative surgical complications and follow-up data were excluded from this study.

### Surgical procedure

All animals were preoxygenated with 100% oxygen. Dogs and cats were premedicated, inducted with medetomidine (10 μg/kg; Domitor, Orion Pharma, Filand) and Zoletil (0.83 mg/kg; Virbac Korea, Korea). Anesthesia was maintained with isoflurane (FORANE, Baxter, USA). Perianal hair was clipped and the area was prepared aseptically for anal sacculectomy. Prior to surgery, the anal sacs were thoroughly lavaged, emptied, and flushed using 0.05% chlorhexidine to reduce the risk of perioperative infection.

For anal sacculectomy, the animals were placed in a padded perineal stand and positioned in sternal recumbency with the tail reflected over their dorsum and secured. A mosquito hemostatic forceps was inserted into the anal sac to grasp the apex of sac. The sac was then exteriorized *via* its orifice. Once the sac was fully everted, the anal sac was simply excised by using a monopolar electrocautery surgical unit (Ethicon Endo-Surgery, Cincinnati, OH, USA). Amoxicillin (20 mg/kg, per oral, twice a day) was administered to all animals for 7 days after surgery to prevent postoperative infection.

### Evaluation of complications

The animals were assessed for postoperative complications by clinicians and owners. The complications associated with the anal sacculectomy were classified as inflammation, excess drainage, acute seroma formation, fecal incontinence and scooting, based on previous publications (6, 9, 11). The evaluations were performed at first day, 1 week, 2 weeks, and 4 weeks after surgery.

## Results

In this study, 17 dogs and 11 cats underwent anal sacculectomy for the surgical treatment of anal sac disease. The breed distribution of the dogs (*n* = 17) was as follows: Bichon (*n* = 3), Dachshund (*n* = 1), French bulldog (*n* = 2), Maltese (*n* = 4), miniature Poodle (*n* = 1), mixed-breed dog (*n* = 4), Pomeranian (*n* = 1), and Pug (*n* = 1). All cats (*n* = 11) were domestic Korean short hair cats. Case signalment including species, body weight, and ages was presented in Table 1. The mean body weight of the dogs was 4.24 ± 0.48 kg (range, 2.2-9.5 kg) and the mean body weight of the cats was 3.45 ± 0.39 kg (range, 2.6–7.2 kg). The mean age of the dogs and cats were 14.62 ± 3.30 and 19.25 ± 7.41 months old, respectively. It indicated that most animals subjected to the anal sacculectomy were small and young adults.

**Table 1:** Case signalment.

**Species**	**Number**	**Body weight (mean ±standard error of mean)**	**Age (months)**
**Dog**	*n =* 17	4.24 ± 0.48 kg (2.2–9.5 kg)	14.62 ± 3.30
**Cat**	*n =* 11	3.45 ± 0.39 kg (2.6–7.2 kg)	19.25 ± 7.41

**Figure 1:**
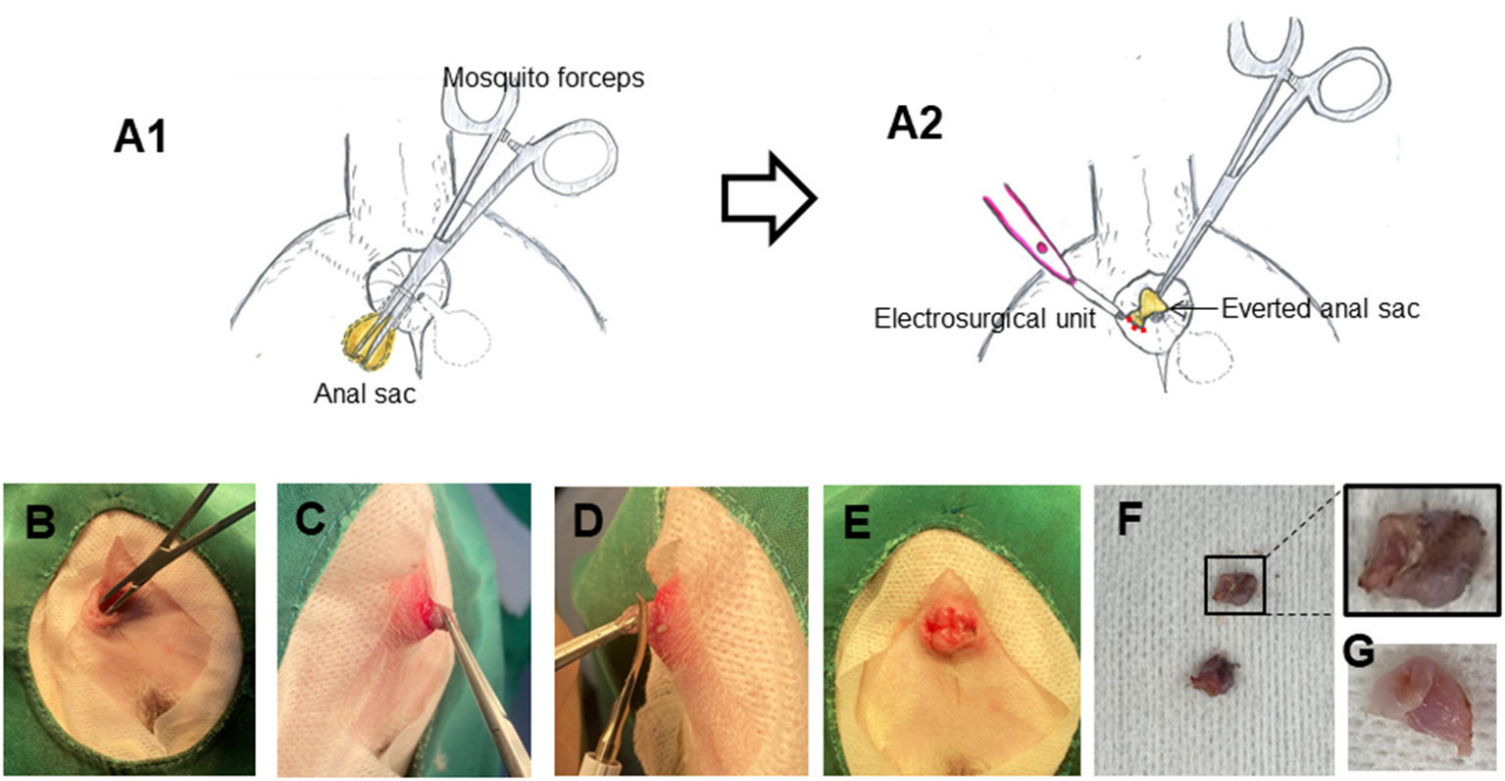
Inside-Out anal sacculectomy. **(A)** Schematic diagrams of Inside-Out anal sacculectomy. **(B–G)** Photographs showing Inside-Out anal sacculectomy in a dog. A mosquito hemostatic forceps was inserted into the anal sac **(B)**, and the anal sac was everted **(C)**. The anal sac was then excised by a monopolar electrocautery surgical unit **(D, E)**. The completely incised anal sacs **(F, G)**.

For Inside-Out anal sacculectomy, the anal sac orifice was firstly widened by using a hemostatic forceps (Halsted mosquito artery forceps; S7-2; straight 12.5 cm, Paramount Co., Pakistan). The forceps was then inserted into the anal sac to grasp the bottom of the anal sac (Figures 1A1, B). The sac was exteriorized and everted fully (Figures 1A2, C). While holding the inverted anal sac with forceps, the anal sac was simply excised by a monopolar electrocautery surgical unit (Figure 1D). The surgical wound after excision remained open, with no suture (Figure 1E; Supplementary movie 1). The operation time to remove both anal sacs ranged from 5 to 10 min in all cases. The excised anal sacs were examined by gross observation to check the entire wall was removed and the muscular attachment to the anal sac. The entire anal sacs were removed without the muscular attachments (Figures 1F, G).

Postoperative complications including inflammation, excess drainage, acute seroma formation, fecal incontinence and scooting were monitored by clinicians and owners at first day, 1 week, 2 weeks, and 4 weeks after surgery (Table 2). There were no major complications requiring surgical intervention. Only 1 dog (mixed-breed dog, 4.78 kg, 6-month-old) showed 2 minor complications; mild fecal incontinence at first day after surgery and scooting behaviors up to one week after surgery. The complications resolved without any additional medical treatments. No complications such as visible inflammation, excess drainage and acute seroma formation were observed in the dogs and the cats given our method.

**Table 2:** Postoperative complications [affected cases / total numbers (*n* = 28)].

**Complications**	**First day after surgery**	**1 week after surgery**	**2 weeks after surgery**	**4 weeks after surgery**
Fecal incontinence	1/28 (1 dog)	0	0	0
Scooting	1/28 (1 dog)	1/28 (1 dog)	0	0
Inflammation	0	0	0	0
Excess drainage	0	0	0	0
Acute seroma formation	0	0	0	0

## Discussion

While conventional anal sacculectomy (open or closed techniques) have been described as effective surgical treatment for anal sac excision, considerable intraoperative and postoperative complications such as fecal incontinence, scooting, inflammation, excess drainage and acute seroma formation have been reported, especially when the anal sacculectomy is performed with an open technique (6, 9, 12). Although the incidence of major complications requiring surgical intervention is relatively low (9), intraoperative complications including iatrogenic trauma to the external anal sphincter muscles, caudal rectal artery and nerve, hemorrhage and rectal laceration, and postoperative complications like fecal incontinence, fistula formation, and surgical site infection are of great concern to clinicians and owners (6, 7, 9, 13). To reduce these complications, the modified closed techniques have been developed. The methods of filling anal sacs with a self-hardening gel or resin, colored yar, umbilical tape or dental acrylic have been introduced in clinics (14). Downs et al. (8) successfully treated 4 dogs with a modified closed anal sacculectomy, in which the balloon of a Foley catheter was applied for dissection of the anal sac (8). Diaz et al. (15) have also suggested a modified balloon-catheter-assisted closed anal sacculectomy (15). Although these methods have been developed to facilitate manipulation and dissection of the anal sac from surrounding tissues, the complication rates of these modified techniques have not been reported.

A review study reported that in 95 dogs received bilateral anal sacculectomy (57 dogs for a closed technique and 38 dogs for an open technique), 3 dogs developed short-term complications such as excess drainage and seroma and 14 dogs developed long-term complications including fecal incontinence, licking and anal stricture, showing that 17 of the 95 dogs (17%) developed postoperative complications (6). Another retrospective study also described that 20 of 62 dogs (32%) developed considerable postoperative complications and small dogs <15 kg body weight experience more complications. Complications were frequently observed in the dogs in which gel was used to distend the anal sac (5). In our method, only 1 dog (1/28 animals, 3%) showed short-term minor complication of mild fecal incontinence and scooting, which resolved within 1 week after surgery without additional medical treatment. Furthermore, when we monitored the dogs and cats for postoperative complications up to maximum 6 months after surgery, no postoperative complications have been observed, suggesting that this technique is a safe method which can reduce postoperative complications, especially in dogs and cats with recurrent anal sac impaction.

In using this technique when a mosquito forceps are inserted into the anal sac to grasp the bottom of sac, care should be taken to ensure that anal sac is not ruptured. Adequate initial everting of anal sac and visualization of lateral margin of the everted anal sacs are crucial in order to remove the entire anal sacs using a monopolar electrocautery surgical unit. Generally, before anal sacculectomy is carried out, inflammation and infection need to be treated medically in order to facilitate complete resection of anal sacs and prevent the rupture of anal sacs during surgery. As anal sacs are supplied from many branches of blood vessels such as rectalis caudalis and perinealis ventralis (16), use of an electrocautery unit in this method is highly recommended to reduce bleeding during cutting the anal sacs. The muscular attachments to the excised anal sacs were not observed by our gross examination (Figure 1G), but the attachments of the internal and external anal sphincter muscles to the external surface of the anal sacs were likely torn or stretched during inversion of the sac, which might cause minor complications including fecal incontinence and scooting behaviors in 1 dog.

In comparison to conventional techniques (6, 7, 9), our “Inside-Out” technique for anal sacculectomy can be performed in a relatively short time (5–10 min) and cause less complications in small dogs and cats. We consider this surgical technique as a good alternative to previously published techniques for cases of non-neoplastic anal sac diseases. In our experience, this method was most easily performed in dogs <1 year of age, or in cats that had more flexible anal sacs than dogs. Therefore, we recommend this method in small dogs <1 year of age, and in cats. Since all the animals that presented to our animal medical center for anal sacculectomy during this study period were small dog breeds (*n* = 17; 2.2–9.5 kg) and cats (*n* = 11; 2.6–7.2 kg), we have not attempted to use this technique in larger dogs (>10 kg) and thus “Inside-Out” anal sacculectomy needs to be investigated in larger dogs.

## Conclusion

We developed a simple and rapid technique for the removal of non-neoplastic anal sacs disease in small dogs and cats, such as anal sac impaction or infection. This procedure was easy, inexpensive and time-saving. This new technique causes far fewer complications than past methods used for small-sized animals. This surgical technique could be a good alternative to conventional techniques.

## Data availability statement

The original contributions presented in the study are included in the article/Supplementary material, further inquiries can be directed to the corresponding author.

## Ethics statement

Ethical review and approval was not required for the animal study because a retrospective study in animal hospital and owners of all animals signed an informed consent form. Written informed consent was obtained from the owners for the participation of their animals in this study.

## Author contributions

Conceptualization: SL, HK, SB, YP, and SJ. Writing—original draft: HK, SL, and CM. Writing—review and editing: CM and SJ. All authors contributed to the article and approved the submitted version.
